# The effect of delirium information training given to intensive care nurses on patient care: quasi-experimental study

**DOI:** 10.7717/peerj.13143

**Published:** 2022-04-08

**Authors:** Fulya Yıldırım, Serpil Türkleş, Hilal Altundal Duru

**Affiliations:** 1 Department of Pediatric Surgery Service, Mersin University Hospital, Mersin, Turkey; 2 Department of Psychiatric and Mental Health Nursing, Mersin University Faculty of Nursing, Mersin, Turkey

**Keywords:** Delirium, Training, Nursing, Patient care, Checklist, Intensive care unit

## Abstract

**Background:**

Training programs aiming to improve delirium diagnosis and management skills increase nurses’ care efficiency and improve patients’ health outcomes. This study was conducted to examine the effect of delirium information training on patient care by intensive care nurses.

**Methods:**

In the research, one group pretest-posttest quasi-experimental design was used. The study sample consisted of 30 nurses working in four intensive care units of a university hospital between November 05, 2018, and February 15, 2019. The Personal Information Form, the Checklist for the Care of the Patient in Delirium, and the Confusion Assessment Scale for the Intensive Care Unit were used to collect the data. Intensive care nurses were provided with information training supported by a training booklet in two sessions of 40 min each.

**Results:**

In the study, according to the Checklist for the Care of the Patient in Delirium, while the pre-test point average of the nurses was 6.17 ± 2.29, the post-test point average had increased to 11.17 ± 1.51 (*p* < 0.001). After the training, it was determined that there was a significant increase in the percentage of nurses who stated that they evaluated and detected whether their patients had pain, hallucinations, and delusions (*p* < 0.001). As a result, it has been determined that providing delirium information training to intensive care nurses positively affects the care of patients with delirium. In addition, it has been determined that with the Confusion Assessment Scale for Intensive Care Unit, nurses can provide the care they need to patients at risk in terms of delirium by identifying delirium.

## Introduction

Delirium is an organic mental syndrome characterized by impaired perception, memory and thought content, increased or decreased physical activity, sleep-wake imbalance, and short-term confusion of the brain affected by many physical, environmental, and physiopathological factors to which the person is exposed (Ely et al., 2001). Delirium is a severe syndrome with a long-term hospitalization period, stay in intensive care, high costs, and increased morbidity and mortality (van den Boogaard et al., 2009; Jackson & Khan, 2015).

Intensive Care Units (ICUs) are unique units designed to serve critically ill patients whose vital functions are at risk or impaired. In the ICU, factors caused by the patient and the characteristics of the intensive care can cause changes in the psychological and mental state of the patient. As a result of these changes, patients in the ICU are at risk for the development of delirium, a condition characterized by impaired consciousness and a cognitive change (Ely et al., 2001; Akıncı et al., 2005). Delirium, which is quite common in critically ill patients, is seen in 30–80% of patients in the ICU (Jackson & Khan, 2015). Studies have reported that patients who develop delirium in the intensive care unit have increased morbidity and mortality, extended hospital stay, and, if not treated, require continuous care due to functional loss after discharge (Ely et al., 2001; Jackson & Khan, 2015). However, delirium is generally overlooked in intensive care patients (Gesin et al., 2012; Panitchote et al., 2015; Rice et al., 2011). Studies have reported that the lack of a regular cognitive evaluation in patients in the ICU, the difficulty of communicating in mechanical ventilation, hypoactive delirium, and the lack of an official assessment tool to monitor delirium affect the delirium recognition rate (Panitchote et al., 2015; Rice et al., 2011).

Nurses hold a vital place in the intensive care unit to evaluate the changes in patients’ conditions and prevent delirium. However, nurses have difficulty recognizing delirium in their patients due to the lack of knowledge about delirium evaluation of the patient, risk factors, and interventions to prevent delirium (Gesin et al., 2012; van de Steeg et al., 2014). Studies have shown that 75% of the nurses working in the ICU cannot detect delirium in the early period (Rice et al., 2011; AbuRuz, 2017), delirium assessment tools are not used effectively, the lack of knowledge about delirium is an essential factor (Rowley-Conwy, 2018), and nurses do not see delirium as a severe syndrome (Xing et al., 2017). Nurses observe patients individually for 24 h and implement their care. Thus, nurses must have sufficient knowledge about delirium, know the risk factors, prevention, and treatment methods to evaluate patients in a holistic manner (Ely et al., 2001). Educational programs to improve delirium diagnosis and management skills increase nurses’ ability to care for patients with delirium and improve patient outcomes (Gesin et al., 2012; van de Steeg et al., 2014). Studies have shown that delirium training increases the knowledge level of nurses (Speed, 2015; Akechi et al., 2010), significantly increases the rate of nurses applying non-pharmacological interventions (Öztürk Birge & Aydın, 2017), after the training on delirium and the scales used to detect delirium, nurses could successfully apply the scales, and that delirium patients could be detected early and start treatment quickly (Harroche, St-Louis & Gagnon, 2014). As a result, as well as the risk of delirium development being extremely high in patients hospitalized in the ICU, patients who are adversely affected by this situation require continuous care. Nurses knowing and managing the risks for delirium development in the ICU can actively cope with this process. Therefore, delirium information training given to intensive care nurses may affect delirium patient care (Gesin et al., 2012; van de Steeg et al., 2014). This research will provide crucial theoretical information to nurses on delirium prevention in intensive care patients, evaluate delirium with an easy measurement tool, and provide appropriate nursing care to an individual with delirium. In this context, this study was conducted to examine the effect of delirium information training on patient care by intensive care nurses. The research hypotheses are given below.

H_0_: Delirium training given to intensive care nurses does not affect patient care.
H_1_: Delirium training given to intensive care nurses has an impact on patient care.

## Materials and Methods

### Design

The research was carried out in one group pretest-posttest quasi-experimental design (Cook, Campbell & Shadish, 2002).

### Setting and participants

The research was planned with nurses working in seven adult ICUs in a university hospital between November 05, 2018–February 15, 2019. Since delirium patients who are not connected to a ventilator could not be reached in three intensive care units including Reanimation, Cardiovascular Surgery and Surgery 2 ICUs between these dates, nurses working in four ICUs, including Internal Medicine-1, Internal Medicine-2, Coronary, Surgery-1 ICU, were included in the study. The ICUs, where the research was done, were tertiary units consisting of 12 beds. Nurses working in these units work in shifts 08-16/16-08, with four or six nurses providing care for three or two patients per shift.

**Figure 1:**
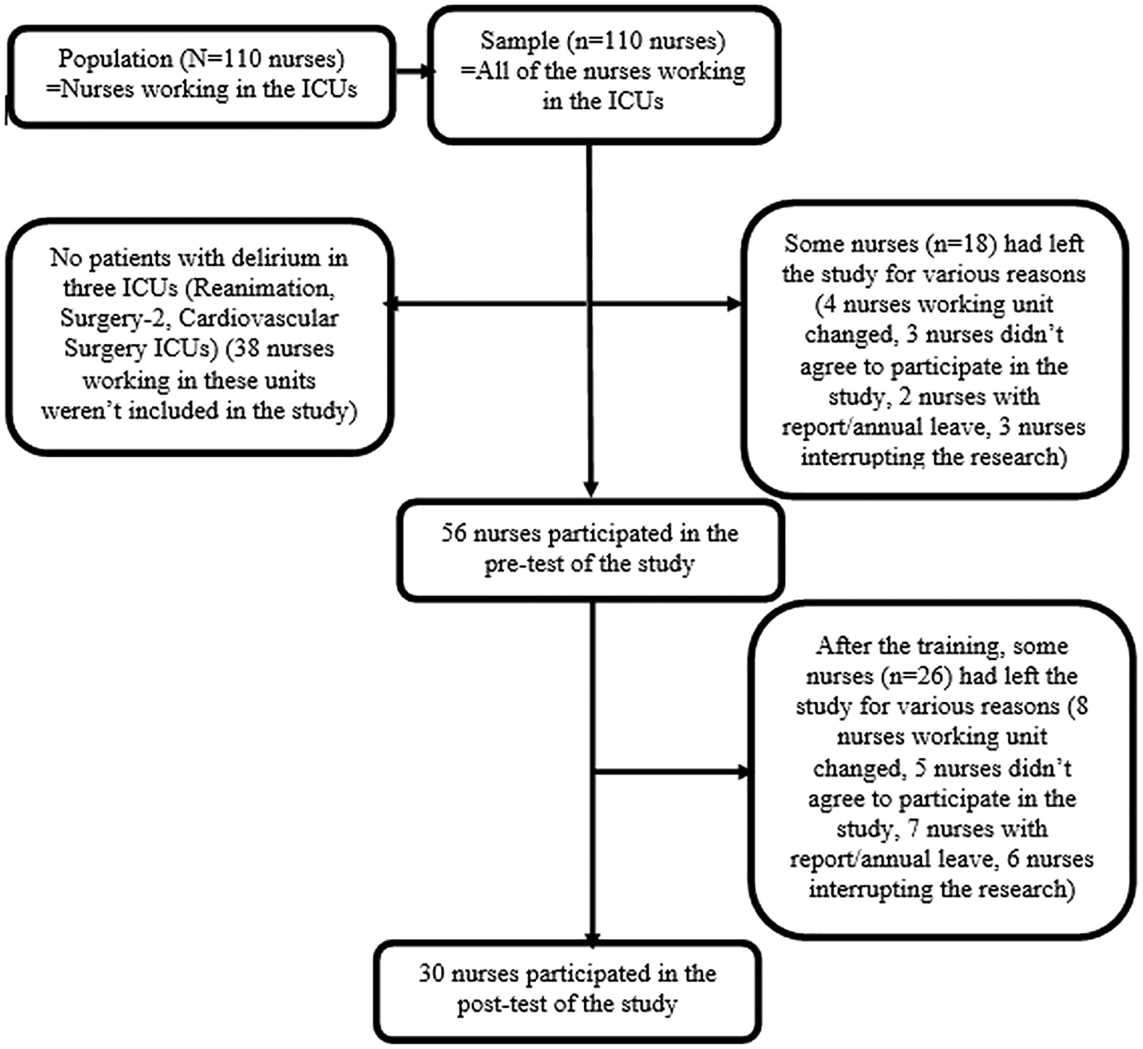
Flow diagram of the study sample.

The study population consisted of all nurses working in seven adult ICUs in a university hospital between November 05, 2018–February 15, 2019 (*N* = 110). Since there was no study similar to our research, the effect size was calculated with our own sample group using the G*Power Program with a power of 0.95 and an error of 0.05, and it was found to be sufficient (effect size = 2.24). According to the calculation, the sample size was determined as five nurses (Faul et al., 2009). However, to provide parametric test conditions, the sample size was aimed to be at least 30 or more (Cook, Campbell & Shadish, 2002). All of the nurses working in the university hospital adult ICU on the dates specified in the study were attempted to be contacted. Since there were no patients with delirium in three ICUs (Reanimation, Surgery-2, Cardiovascular Surgery ICU) on the specified dates, 38 nurses working in these units were not included in the study. Therefore, a total of 72 nurses working in the remaining ICUs (Internal Medicine-1, Internal Medicine-2, Surgery-1, and Coronary ICU) constituted the sample of the study. Later, among these 72 people, some had left the study for various reasons (four nurses working unit changed, three nurses did not agree to participate in the study, two nurses went on sick/annual leave, three nurses interrupted the research), and 56 nurses participated in the pre-test of the study. After the training, the number of nurses participating in the post-test decreased to 30 due to the same reasons (eight nurses working unit changed, five nurses did not agree to participate in the study, seven nurses went on sick/annual leave, six nurses interrupted the research) (Fig. 1).

### Data collection

The study data was collected using “Personal Information Form” (Gesin et al., 2012; Öztürk Birge & Aydın, 2017) and “Checklist for the Care of the Patient in Delirium” (Akıncı et al., 2005; Gesin et al., 2012; Öztürk Birge & Aydın, 2017) which were developed in line with the literature knowledge. In addition, the “Confusion Assessment Scale for the Intensive Care Unit (CAM-ICU)” (Ely et al., 2001; Akıncı et al., 2005) was used.

Personal Information Form: This form consists of open-ended and multiple-choice questions developed by researchers after literature reviews. It consists of nine questions about nurses’ individual characteristics such as gender, age, education, and duration of work in the intensive care unit (Gesin et al., 2012; Öztürk Birge & Aydın, 2017).

Checklist for the Care of the Patient in Delirium: This checklist was prepared by the researchers in line with the literature reviews to determine the nursing care applied to the patient with delirium. The Checklist for the Care of the Patient in Delirium consists of 13 questions (Akıncı et al., 2005; Gesin et al., 2012; Öztürk Birge & Aydın, 2017). The control of each statement in the checklist is either “Done” or “Not Done”. In order to evaluate the conformity of the checklist in terms of scope and content, expert opinions of four faculty members who are experts in their fields were consulted. The scoring of the checklist was done *via* the researchers as a result of their observations by giving 1 point to those marked “Done”, “0” to those marked “Not Done”. The lowest and highest scores to be obtained from the scale are 0 and 13, respectively.

Confusion Assessment Scale for the Intensive Care Unit (CAM-ICU): The validity and reliability study of the scale, which was developed by Ely et al. (2001), was verified by Akıncı et al. (2005) so that it could be used in Turkey. In order to be diagnosed with delirium (CAM-ICU positive), the participant must show characteristics 1, 2, 3, or 4. CAM-ICU has high reliability and specificity in the original validation study, including adults in the medical and coronary ICU. For CAM-ICU, 95% sensitivity and 98% specificity were defined. CAM-ICU consists of four items. In the scale below, delirium is diagnosed when the first two items are positive, and either the third or fourth items are positive (Ely et al., 2001; Akıncı et al., 2005).

### Interventions

The research was carried out in six stages:

1st Stage: After the approval of the ethics committee and the institution’s permission, the nurses working in the ICU were informed about the name, purpose, application method, and duration of the research before the training. After the verbal consent of the nurses was given, they were asked to fill in a “Personal Information Form,” and then the researcher took this form back.

2nd Stage: CAM-ICU was applied by the researcher and the observer to detect delirium in patients with delirium risk (who exhibit agitated, hyperactive, and disorientated behaviors) in four ICUs included in the scope of the study. The observer is an expert in psychiatric nursing and studying for a doctorate in this field. The patients were evaluated with CAM-ICU during the day shift between 10:00 and 12:00 and during the night shift between 20:00 and 22:00. In the evaluation of the patients, their previous 24-h condition was taken into account.

3rd Stage: While the nurse was providing care to the patient diagnosed with delirium, the “Checklist for the Care of the Patient in Delirium” was observed and filled by the researcher. While filling the checklist, the checklist was simultaneously filled by the observer to maintain the study’s reliability. The nurses were not informed that they were being observed by the researcher and the observer to maintain their natural approach to the patient during their interventions. At the beginning of the study, voluntary consent forms were obtained from the nurses. The researchers stated that they would observe the nurses doing their routine care at the bedside during any visit to the clinic. For this reason, the researchers did not remind that “we are observing you right now” so that the nurses maintain their natural behavior while giving care. The researcher and the observer observed the nurse while giving care to her patient, keeping a certain distance between them. After the observation, the nurses were informed about the training days and hours.

4th Stage: The researcher gave training on “Delirium and Nursing Approach in Intensive Care Units” to the nurses within 3 weeks per the work plan of the nurses. This training was prepared by the researchers in line with the current literature and based on the results of the evidence-based research. The training content included delirium symptoms, risk factors, frequency and prevalence, types, evaluation, diagnostic criteria, the situations of nurses’ realization that the patient is in delirium, prevention, measurement tools, treatment, and nursing approaches. The training was given to five groups of 10–12 people in two sessions in the ICU training room. The first session lasted for 40 min, and theoretical information was given in the barcovision with a slide presentation with lectures, discussion, and question-answer techniques. After a 15-min break, in the second session, a video was shown about the care of individuals with delirium, and interactive group work was done. This interactive group work consisted of Kahoot games, brainstorming, discussions of cases, and nurses’ experiences with their patients with delirium. At the end of the training, the “Delirium and Nursing Approach in Intensive Care Units Training Booklet” prepared by the researchers was given to the nurses.

5th Stage: Three weeks after the training, CAM-ICU was applied to the patients who had delirium risk in the ICU where nurses worked, by the researcher and the observer, and it was determined whether they were in delirium or not.

6th Stage: After determining the patients with delirium, the “Checklist for the Care of the Patient in Delirium” was filled in by the researcher in the presence of an observer while the nurse was providing care to the patient diagnosed with delirium. The application stages of the research were shown in Table 1.

**Table 1:** The application stages of the research.

Stage	The applications
1st Stage	Getting verbal consent of nurses Nurses to fill the “Personal Information Form”.
2nd Stage	In order to detect delirium in patients with delirium risk; Application of CAM-ICU by the researcher and the observer.
3rd Stage	While the nurse was providing care to the patient diagnosed with delirium; Filling the “Checklist for the Care of the Patient in Delirium” by the researcher and the observer. Next; Informing the nurses about training days and hours.
4th Stage	Within a total of 3 weeks; Training of “Delirium and Nursing Approach in Intensive Care Units” to the nurses by the researcher.
5th Stage	In order to detect delirium in patients with delirium risk observed 3 weeks after the training; Application of CAM-ICU by the researcher and the observer.
6th Stage	While the nurse was providing care to the patient diagnosed with delirium; Filling the “Checklist for the Care of the Patient in Delirium” by the researcher and the observer.

### Data analysis

The data obtained from the study were analyzed using the Statistical Package for the Social Sciences (SPSS) 20.0 for Windows statistical program. Descriptive statistical methods (mean, standard deviation, frequency, minimum and maximum value) were used to evaluate the data. Whether the data showed a normal distribution was examined with the Shapiro–Wilk test. Pearson’s chi-square test was used to compare categorical variables between groups. Wilcoxon Signed Ranks Test was used to compare the pre-test and post-test point averages. The results were evaluated in a confidence interval at 95% and by the significance level of *p* < 0.05.

### Ethical consideration

The study was conducted according to the guidelines of the Declaration of Helsinki and approved by the Institutional Review Board (or Ethics Committee) of Mersin University Clinical Research Ethics Committee (protocol code 17/379 and date of approval 03.10.2018). All nurses gave their verbal and written informed consent for inclusion before they participated in the study. Informed consent was obtained from all subjects involved in the study. “The Checklist for the Care of the Patient in Delirium” was not shown to the nurses to not affect the nurses in the pre-test and post-test. In order to prevent the researcher and the observer from being affected by each other, each of them filled out the “Checklist for the Care of the Patient in Delirium” separately, and after the test, the researcher and the observer combined their observations with a joint decision.

### Limitations

This study had some limitations. The main limitation of this study was conducted in one group with no control group and non-randomization. Another limitation of this study is that there were no cases in three ICUs where the study was conducted, the number of cases was low, and the nurses were not regularly involved in every phase of the study. As a strength of the study, this research provided the opportunity to observe nurses before-after the training for a long period. However, in ICUs with open section design, nurses’ attempts to provide care to patients with delirium may have been affected due to the interaction of nurses with each other. Finally, the Checklist for the Care of the Patient in Delirium, used in data collection, was compiled from international research results. Therefore, this form was not subjected to a validity and reliability analysis. However, this aspect of the research was strengthened by ensuring that the two observers filled out the form separately. Finally, based on all these limitations, these findings are not generalizable to other intensive care nurses.

## Results

The age range of the nurses participating in the study was 21–48, and their mean age was 28.73 ± 6.05. It was determined that 66.7% of the nurses were women, 73.3% had bachelor’s degrees, and 53.3% were single (Table 2).

**Table 2:** Distribution of nurses’ socio-demographic characteristics.

Characteristics	Minimum–Maximum	$\bar{x}$ ± sd
Age	21–48	28.73 ± 6.05
	*n*	%
Gender		
Female Male	20 10	66.7 33.3
Educational status		
High school Associate Degree Bachelor Graduate	6 1 22 1	20 3.3 73.3 3.3
Marital status		
Single Married	16 14	53.3 46.7

**Note:**

$\bar{x}$, mean; sd, standard deviation; *n*, number; %, percent.

The working time of the nurses participating in the study in the ICU was between 1 and 220 months, and the mean working time was 48.93 ± 49.62 months. The number of patients that nurses provided care in a shift varied between 2–6 and the mean of it was 3.23 ± 0.73. 16.5% of the nurses worked in Coronary, 53.3% in Internal Medicine, and 30% in Surgery ICU. It was found that 93% of the nurses participating in the study had not received training on delirium before. When the status of nurses’ previous delirium patient caregiving was examined, it was determined that all of the nurses gave care to delirium patients (Table 3).

**Table 3:** Nurses’ working status and distribution of delirium characteristics.

Characteristics	Minimum–Maximum	$\bar{x}$ ± sd
The working time in the ICU (month)	1–220	48.93 ± 49.62
Number of patients given care in a shift	2–6	3.23 ± 0.73
	*n*	%
Employed ICU		
Coronary ICU Internal Medicine ICU Surgery ICU	5 16 9	16.7 53.3 30
Previous delirium training status		
Yes No	2 28	6.7 93.3
Previous delirium patient care status		
Yes No	30 0	100 0

**Note:**

ICU, Intensive Care Unit; $\bar{x}$, mean; sd, standard deviation; *n*, number; %, percent.

When the mean scores of the nurses regarding the intervention checkpoints before and after the training were compared, it was determined that there was a significant difference in all of the intervention checkpoints mean scores after the training (*p* < 0.001; *p* < 0.01; *p* < 0.05). After the training, nurses’ application of intervention checkpoints increased (Table 4).

**Table 4:** Comparison of the nurses’ application of intervention control points before and after the training.

Intervention control points	Before training				After training				Significance level
	Performed		Not performed		Performed		Not performed		
	*n*	%	*n*	%	*n*	%	*n*	%	
Introduced himself/herself to the patient by saying his/her name.	3	10	27	90	26	86.7	4	13.3	x^2^ = 35.308^a^ df = 1 *p* < 0.001***
Evaluated/provided the patient’s orientation to the ground.	22	73.3	8	26.7	30	100	0	0	x^2^ = 9.231^a^ df = 1 *p* = 0.002**
Evaluated/provided the patient’s personal orientation.	24	80	6	20	30	100	0	0	x^2^ = 6.667^a^ df = 1 *p* = 0.010*
Evaluated/provided the patient’s time orientation.	12	40	18	60	29	96.7	1	3.3	x^2^ = 22.259^a^ df = 1 *p* < 0.001***
Questioned/observed whether the patient had pain or not.	10	33.3	20	66.7	23	76.7	7	23.3	x^2^ = 11.380^a^ df = 1 *p* = 0.001**
Reduced the stimuli around the patient.	3	10	27	90	12	40	18	60	x^2^ = 7.200^a^ df = 1 *p* = 0.007**
Raised the borders of the bed.	29	96.7	1	3.3	30	100	0	0	x^2^ = 1.017^a^ df = 1 *p* = 0.313
Spoke slowly and clearly with the patient.	26	86.7	4	13.3	30	100	0	0	x^2^ = 4.286^a^ df = 1 *p* = 0.038*
Assessed whether the patient had hallucinations.	3	10	27	90	20	66.7	10	33.3	x^2^ = 20.376^a^ df = 1 *p* < 0.001***
Evaluated whether the patient had delusions or not.	3	10	27	90	20	66.7	10	33.3	x^2^ = 20.376^a^ df = 1 *p* < 0.001***
Listened to the patient and allowed him/her to express his feelings.	24	80	6	20	30	100	0	0	x^2^ = 6.667^a^ df = 1 *p* = 0.010*
Gaved precise and direct commands to the patient.	21	70	9	30	30	100	0	0	x^2^ = 10.588^a^ df = 1 *p* = 0.001**
Daytime hours enabled the environment to be bright and at night to be dimmer.	5	16.7	25	83.3	25	83.3	5	16.7	x^2^ = 26.667^a^ df = 1 *p* < 0.001***

**Notes:**

a, Pearson’s Chi-Square test; df, degrees of freedom; *p*, significance level; *n*, number; %, percent.

* *p* < 0.05.

** *p* < 0.01.

*** *p* < 0.001.

Before the training, 30% of the nurses stated that they evaluated whether the patients had pain, and 13.3% reported that they detected their patients’ pain. After the training, 76.7% of the nurses stated that they evaluated whether the patients had pain, and 60% stated that they detected their patients’ pain (*p* < 0.001). Before the training, 10% of the nurses stated that they evaluated whether the patients hallucinated, and none of them could detect hallucinations with their patients. After the training, 66.7% of the nurses stated that they evaluated whether the patients hallucinated, and 30% stated that they detected their patients’ hallucinations (*p* < 0.001). Before the training, 13.3% of the nurses stated that they assessed whether the patients had delusions/delirium, and 3.3% reported that they detected their patients’ delusions/delirium. After the training, 66.7% of the nurses stated that they evaluated whether the patients had delusions, and 30% stated that they detected their patients’ delusions (*p* < 0.001) (Table 5).

**Table 5:** Comparison of the responses of the nurses to the intervention control questions before and after the training.

Intervention control points	Before training				After training				Significance level
	Evaluated				Evaluated				
	In the patient				In the patient				
	Yes		No		Yes		No		
	*n*	%	*n*	%	*n*	%	*n*	%	
Pain assessment status in the patient	4	13.3	5	16.7	18	60	5	16.7	x^2^ = 15.909^a^ df = 2 *p* < 0.001*
Hallucination evaluation status in the patient	0	0	3	10	9	30	11	36.7	x^2^ = 21.382^a^ df = 2 *p* < 0.001*
Delusion assessment status in the patient	1	3.3	3	10	9	30	11	36.7	x^2^ = 18.083^a^ df = 2 *p* < 0.001*

**Notes:**

a, Pearson’s Chi-Square test; df, degrees of freedom; *p*, significance level; *n*, number; %, percent.

* *p* < 0.001.

According to the Checklist for the Care of the Patient in Delirium, the nurses’ mean scores on the pre and post-tests were 6.17 ± 2.29 and 11.17 ± 1.51 respectively. It was determined that the difference between pre-test and post-test mean scores was statistically significant (*p* < 0.001) (Table 6).

**Table 6:** Comparison of the nurses’ pretest–posttest mean scores according to the checklist for the care of the patient in delirium.

		$\bar{x}$ ± sd	Significance level
Checklist for the Care of the Patient in Delirium	Pre-test	6.17 ± 2.29	*z* = −4.756^a^ *p* < 0.001*
	Post-test	11.17 ± 1.51	

**Notes:**

a, Wilcoxon Signed Ranks Test; *z*, Wilcoxon Signed Ranks Test result value; $\bar{x}$, mean; sd, standard deviation; *p*, significance level.

* *p* < 0.001.

## Discussion

In this study, while providing care to their patients with delirium after the training, a significant increase was determined in nurses’ application of intervention checkpoints. In addition, a significant increase was found in nurses’ evaluation and detection of pain, hallucinations, and delusions while caring for patients with delirium after the training. These results are supported by existing literature. In the study in which Öztürk Birge & Aydın (2017) provided non-pharmacological interventional training for delirium to intensive care nurses, it was determined that in the post-training phase, nurses’ practice of reducing noise in the unit, removing unnecessary equipment from the environment, and supporting orientation with a calendar and clock increased significantly. It was determined that nurses’ avoidance of dividing sleep hours with treatment hours, providing dim lighting in the night unit environment, and applying practices to support daytime alertness of the patient increased significantly (Öztürk Birge & Aydın, 2017). Şahin (2019) conducted a descriptive study to investigate the knowledge and attitudes of nurses about delirium using a questionnaire with 420 nurses working in a university hospital. It was found that 76.4% of the nurses within the scope of the study had individuals who hallucinated, illusions, fear, and anxiety in the patients they care for, and 79.5% of them applied place, person, and time orientation to their patients (Şahin, 2019).

In this study, it was found that the difference between the pre-test and post-test point averages of nurses according to the Checklist for the Care of the Patient in Delirium was statistically significant. This result confirms the hypothesis that “Delirium training given to intensive care nurses impacts patient care”. Providing informative training to intensive care nurses in two 40-min sessions, supported by the training booklet, positively affects the care of patients with delirium. This study determined that nurses could provide the care they need to patients at risk of delirium by determining delirium with CAM-ICU in intensive care patients. In addition, through the observation method, the application status of the intervention points in the checklist while providing care to their delirium patients was evaluated. Interventional training programs to improve the ability to diagnose and manage delirium increase nurses’ efficiency, and improve patient care outcomes (Gesin et al., 2012; Wand et al., 2014). Interventional training programs for nursing care in delirium are effective approaches to reduce delirium incidence, severity, and duration (Barr et al., 2013; Irwin et al., 2013). In the literature, a study evaluating the nursing care of a patient with delirium with a checklist was found. Öztürk Birge & Aydın (2017) investigated the effect of non-pharmacological training on delirium identification and intervention strategies of intensive care nurses. It was found that the training given increases the rate of delirium recognition by nurses and decreases the delirium incidence in the ICU. In addition, it was determined that ICU nurses applying non-pharmacological interventions to prevent delirium increased after the training (Öztürk Birge & Aydın, 2017). These are similar to our research results. In the studies conducted in Turkey, the conduct of the intervention studies with nurses to prevent delirium stands out. Çavuşoğlu (2019) proved that the management of environmental stimuli in the ICU could reduce the risk of developing delirium in patients. Topçu (2019) found that non-pharmacological arrangements (reduction of “light and sound”) in ICU reduce the incidence of delirium in patients and that the decrease in noise level positively affects the sleep quality of the patient. Elibol (2015) found that nursing approaches to prevent delirium risk factors effectively reduced the severity and incidence of delirium in elderly patients who underwent orthopedic surgery in her study.

When the international literature is examined, studies show that delirium information training is effective in the knowledge level of nurses and the evaluation and management of delirium in patients (Gesin et al., 2012; Speed, 2015; Akechi et al., 2010; Harroche, St-Louis & Gagnon, 2014; Wand et al., 2014; Archer, 2017). It has been reported that the training given about delirium and delirium screening tools has an impact on nurses’ ability to diagnose delirium clinically and to use a standard delirium scale correctly (Hickin, White & Knopp-Sihota, 2017; Ramoo et al., 2018; Smith et al., 2017). In one study, a significant increase in nurses’ knowledge of delirium and the diagnosis of delirium was reported with web-based delirium information training (McCrow, Sullivan & Beattie, 2014). These results are supported by existing literature. Unlike our study findings, one study found that the delirium knowledge levels of nurses remained the same at the end of the online learning module and simulation-supported training (Smith et al., 2017). At the end of another simulation supported delirium information training program, it was concluded that delirium screening and documentation in nurses were still weak (Lieow et al., 2019). This difference may arise from the training place (online or face-to-face) and the sample of the research (advanced practice nurse or ICU nurse).

## Conclusions

With this study, it was determined that giving delirium information training to intensive care nurses positively affected the care of patients with delirium. After the training, it was found that nurses’ application of interventions at intervention check points increased while providing care to their patients with delirium. In addition, after the training, it was found that there was a significant increase in the evaluation and detection of pain, hallucinations, and delusions in their patients. At the same time, it was determined that with CAM-ICU, nurses could provide the care they need to patients at risk in delirium by determining delirium in intensive care patients. Per these results it is recommended that:

- Delirium informing training is given regularly, supported by the educational booklet, in ICUs within the scope of the orientation of nurses,
- evidence-based programs for early diagnosis of delirium and effective management of delirium in the ICU are developed, implemented, and evaluated,
- nurses add CAM-ICU, an easy–to-use measurement tool with validity and reliability, to observation forms for the early diagnosis of delirium in their patients and to apply it regularly to their patients,
- measurable guidelines are established in line with evidence-based quality standards for delirium development in the ICU and nursing care in the ICU be evaluated at regular intervals in line with these guidelines,
- follow-up studies are conducted with a larger sample to determine whether the improvement in delirium detection will continue in the long term.

## Supplemental Information

10.7717/peerj.13143/supp-1 Supplemental Information 1 Questionnaire (English).

10.7717/peerj.13143/supp-2 Supplemental Information 2 Questionnaire (Turkish).

10.7717/peerj.13143/supp-3 Supplemental Information 3 Raw data (English).

10.7717/peerj.13143/supp-4 Supplemental Information 4 Raw data (Turkish).

## Additional Information and Declarations

### Data Availability

The following information was supplied regarding data availability: The raw measurements are available in the Supplemental Files.
