# Supplementary material for: The effect of delirium information training given to intensive care nurses on patient care: quasi-experimental study
Source: PeerJ. 2022 Apr 8;10:e13143. doi: 10.7717/peerj.13143 (PMC8997191; doi:10.7717/peerj.13143)

**Personal Information Form**

**1.Age:**

**2.Gender**

- Female
- Male

**3.Educational status**

- High school
- Associate Degree
- Bachelor
- Graduate

**4. Marital status**

- Married
- Single

**5.** **Intensive Care Unit where you work**

- Coronary ICU
- Internal Medicine ICU
- Surgery ICU
- Reanimation Intensive Care
- Cardiovascular Surgery Intensive Care

**6.** **Your Service Period in Intensive Care…..…..Year ……..Month**

**7.** **How many patients do you care for in a shift? (Please write)…………………...**

**8. Have you received training on delirium before?**

( ) Yes (Please write) ……………………………………………………….….

( ) No

**9. Have you ever had a patient in your unit who went into delirium?**

( ) Yes (Please write how many patients there are)…………………………………. ..

( ) No

**The Checklist for the Care of the Patient in Delirium**

| **Intervention control points** | **Performed** | **Not performed** |
| --- | --- | --- |
| 1. He/she introduced himself to the patient, saying his name. |  |  |
| 2. Assessed/provided the patient's ground orientation (e.g. ask/tell where it is located). |  |  |
| 3. Assessed/provided the patient's person orientation (For example: asking name, asking age, calling by name). |  |  |
| 4. Assessed/provided the patient's time orientation (For example: asking/reminding date, day and time). |  |  |
| 5. He/she questioned/observed whether the patient had pain. |  |  |
| 6. Reduced the stimuli around the patient (such as sound, noise). |  |  |
| 7. He/she lifted the borders of the bed. |  |  |
| 8. He/she spoke slowly and clearly to the patient. |  |  |
| 9. He/she evaluated whether the patient had hallucinations. |  |  |
| 10. He/she evaluated whether the patient had delusions. |  |  |
| 11. He/she listened to the patient and allowed him to express his feelings. |  |  |
| 12. He/she gave precise and direct commands to the patient. |  |  |
| 13. Daytime hours, on the other hand, allowed the environment to be bright, and at night, the environment to be dim. |  |  |

1. **Did your patient have pain?**

- Yes
- No
- Not evaluated

1. **Did your patient have hallucinations?**

- Yes
- No
- Not evaluated

1. **Did your patient have delusions?**

- Yes
- No
- Not evaluated


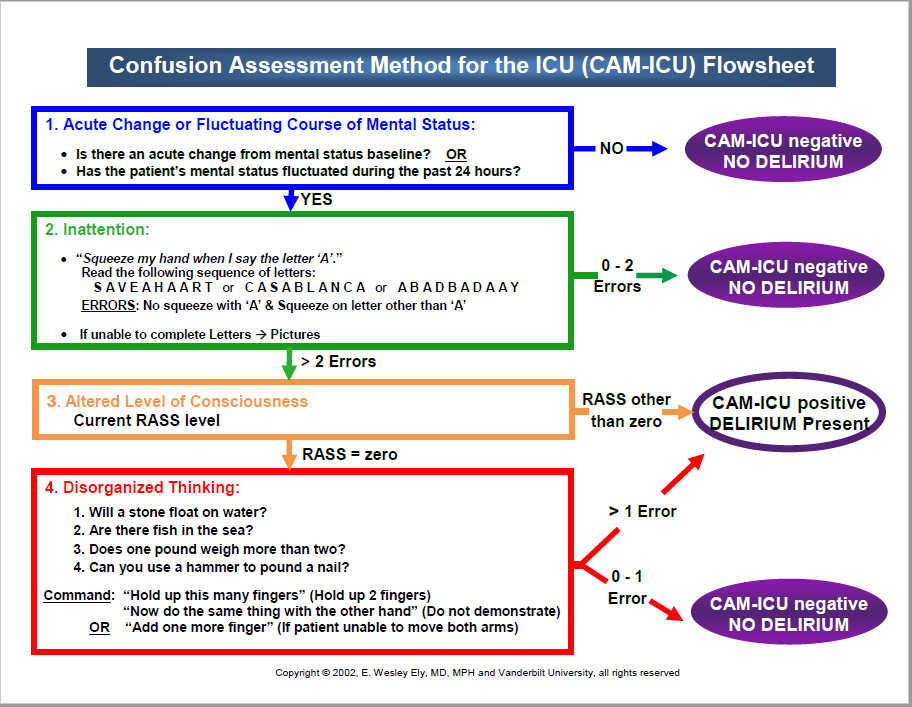

Supplement: Supplemental Information 1 [file peerj-10-13143-s001.docx]
