# Supplementary material for: The effect of delirium information training given to intensive care nurses on patient care: quasi-experimental study
Source: PeerJ. 2022 Apr 8;10:e13143. doi: 10.7717/peerj.13143 (PMC8997191; doi:10.7717/peerj.13143)
Supplement: Supplemental Information 2 [file peerj-10-13143-s002.docx]

**Kişisel Bilgi Formu**

1-Yaşınız…..

2- Cinsiyetiniz:

( ) Kadın

( ) Erkek

3- Eğitim düzeyiniz:

( ) Lise

( ) Önlisans

( ) Lisans

( ) Lisansüstü

4- Medeni Durumunuz:

( ) Evli

( ) Bekar

5- Çalıştığınız Yoğun Bakım Ünitesi

( ) Koroner Yoğun Bakım

( ) Dahiliye Yoğun Bakım

( ) Cerrahi Yoğun Bakım

( ) Reanimasyon Yoğun Bakım

( ) Kalp Damar Cerrahi Yoğun Bakım

6- Yoğun Bakımdaki Hizmet Süreniz…..…..Yıl ……..Ay

7- Bir vardiya süresinde kaç hastaya bakım veriyorsunuz? (Lütfen yazınız)…………………...

8- Daha önce delirium konusunda eğitim aldınız mı?

( ) Evet (Lütfen yazınız)…………………………………………………………….….

( ) Hayır

9- Biriminizde deliryuma giren bir hastanız oldu mu?

( ) Evet (Lütfen kaç hasta olduğunu yazınız)……………………..................................

( ) Hayır

**Deliryumdaki Hastanın Bakımına İlişkin Kontrol Listesi**

| **Girişim Kontrol Noktaları** | **Yaptı** | **Yapmadı** |
| --- | --- | --- |
| 1. İsmini söyleyerek hastaya kendisini tanıttı. | ( ) | ( ) |
| 1. Hastanın yer oryantasyonunu değerlendirdi/sağladı (Örneğin nerede bulunduğunu sormak/söylemek) | ( ) | ( ) |
| 1. Hastanın kişi oryantasyonunu değerlendirdi/sağladı (Örneğin: ismini sormak, yaşını sormak, ismiyle hitap etmek). | ( ) | ( ) |
| 1. Hastanın zaman oryantasyonunu değerlendirdi/sağladı (Örneğin: tarih, gün ve saati sormak/hatırlatmak). | ( ) | ( ) |
| 1. Hastanın ağrısının olup olmadığını sorguladı/gözlemledi | ( ) | ( ) |
| 1. Hastanın çevresindeki uyaranları azalttı. (ses, gürültü gibi). | ( ) | ( ) |
| 1. Yatağın kenarlıklarını kaldırdı. | ( ) | ( ) |
| 1. Hasta ile yavaş ve anlaşılır bir şekilde konuştu. | ( ) | ( ) |
| 1. Hastanın halüsinasyonlarının olup olmadığını değerlendirdi. | ( ) | ( ) |
| 1. Hastanın sanrılarının olup olmadığını değerlendirdi. | ( ) | ( ) |
| 1. Hastayı dinledi ve duygularını ifade etmesine izin verdi. | ( ) | ( ) |
| 1. Hastaya kesin/net ve doğrudan komutlar verdi. | ( ) | ( ) |
| 1. Gündüz saatleri ise ortamın aydınlık olmasını ve gece ise ortamın daha loş olmasını sağladı. | ( ) | ( ) |

**1. Hastanızın ağrısı var mıydı?**

( ) Var

( ) Yok

( ) Değerlendirmedim

**2. Hastanızın halüsinasyonu/varsanısı var mıydı?**

( ) Var

( ) Yok

( ) Değerlendirmedim

**3. Hastanızın sanrısı/hezeyanı var mıydı?**

( ) Var

( ) Yok

( ) Değerlendirmedim

**Yoğun Bakım Ünitesinde Konfüzyonun Değerlendirme Ölçeği (CAM-ICU)**

1. Hastanın bilinç durumunda ani değişiklik oldu mu?

-Bilinci açılıp, kapandı mı?

-Başlangıçtan farklı ani bilinç değişikliği oldu mu?

-Son 24 saatte bilinci açılıp, kapandı mı?

-Uygunsuz davranışının düzelip, kötüleştiği oldu mu?

-Sedasyonskalası veya koma skalası (Glasgow koma skalası) değerleri son 24 saat içinde değişti mi?

1. Dikkat bozukluğu

-Hasta dikkatini toplamakta zorluk çekiyor mu?

-Hastanın dikkatini sürdürmekte veya başka yöne kaydırmakta sıkıntısı var mı?

-Dikkat değerlendirme muayenesinde başarılı oldu mu?

-Şimdi size harfleri okuyacağım. Her A harfini duyduğunuzda elimi sıkınız (bir harfi bir saniyede okuyun). L T P E A O A I C T D A L A AA N I A B F S A M R Z E O A D P A K L A U C J T O E A B A A Z Y F M U S A H E V A A R A T

Sekizden fazla doğru cevap verdi mi?

1. Düşünce organizasyonunun bozulması

-Hasta ekstübe ise, hastanın konuşması, düşünce içeriği, akışı ve organizasyonu bozulmuş mu, düşünce içeriği konudan konuya atlama şeklinde mi?

-Hasta ventilatörde ise şu sorulara cevap verebiliyor mu?

• Taş suda yüzer mi?

• Denizde balık var mı?

• 1 kg 2 kg’dan daha mı ağırdır?

• Çivi çakmak için çekiç kullanabilir misiniz?

-Hasta soruları izleyebiliyor, aşağıdaki komutlara uyabiliyor mu?

• Düşüncelerinizde karışıklık, düzensizlik var mı?

• Bu kadar parmağınızı kaldırın (muayene eden, hastanın görebileceği mesafede iki parmağını kaldırır)

• Aynı hareketi öbür elinizle yapın

1. Kapanmış bilinç düzeyi

-Aşırı alertLetarjik (uykulu fakat hemen uyandırılabiliyor, çevresinde olup bitenlerin bazılarının farkında değil)

-Stupor (zorla veya tekrarlayan stimuluslarla uyandırılıyor, çevresinde olup bitenlerin çoğunun veya hiçbirisinin farkında değil)

-Koma (uyandırılamıyor)
